# CTX-M β-lactamase–producing *Escherichia coli* in Long-term Care Facilities, France

**DOI:** 10.3201/eid1009.030969

**Published:** 2004-09

**Authors:** Najiby Kassis-Chikhani, Sophie Vimont, Karine Asselat, Christophe Trivalle, Bérédia Minassian, Christian Sengelin, Valérie Gautier, Danièle Mathieu, Elisabeth Dussaix, Guillaume Arlet

**Affiliations:** * Hôpital Paul Brousse, Villejuif, France;; † Hôpital Tenon, Paris, France;; ‡ Université Paris VI, Paris, France

**Keywords:** letter, outbreak, multidrug resistant, infections, long-term care facilities, ESBL-producing, E. coli, CTX-M

**To the Editor:** In long-term care facilities, most endemic infections affect respiratory and urinary tracts, as well as skin and soft tissues (*1**–**3*). Infection and colonization by antimicrobial-resistant organisms, in particular those producing plasmid-mediated extended-spectrum β-lactamases (ESBL), are common in long-term care facilities (*4*). Since 1984, ESBL-producing *Enterobacteriaceae* have spread among French hospitals; within Parisian public hospitals (Assistance Publique, Hôpitaux de Paris), ESBL-producing *Escherichia coli* is the most frequent species found, representing 49.5% of 220 *Enterobacteriaceae* isolated in 2002, mostly in urinary tract infections (*5*). Among ESBL-producing *Enterobacteriaceae*, CTX-M–type β-lactamases confer a higher level of resistance to cefotaxime and ceftriaxone than to ceftazidime. CTX-M–producing strains are endemic in Latin America, Japan, and certain parts of Eastern Europe; in contrast, these strains are emerging in France, Western Europe, and the United States (*6*). We report the first documented outbreak of CTX-M–producing *E. coli* infection in a long-term care facility in France. Our hospital is an 800-bed institution with 300 beds for long-term patients distributed among three units located in two buildings. The outbreak occurred in a 35-bed unit and involved 26 of 47 hospitalized patients from October 2001 to March 2003. This facility hosts patients for extended periods of time or permanently. The index case was identified in October 2001; the patient had a urinary tract infection attributable to an ESBL-producing *E. coli*, which showed resistance patterns not previously found in our hospital. Three new cases were detected within the following 2 months, and all patients had urinary tract infection with the same pattern of resistance. In January 2002, patients were screened for ESBL-producing strains by rectal swabbing and urine culture. The results showed *E. coli* with a high level of resistance to amoxicillin and ticarcillin (MIC > 128 µg/mL), partial restoration of susceptibility to these agents by addition of clavulanic acid (MIC = 16–32 µg/mL), and higher resistance to cefotaxime (MIC > 128 µg/mL) than to ceftazidime (MIC = 32–64 µg/mL.) A cephalosporin/co-amoxiclav synergy test was positive, which suggests a CTX-M ESBL. Strains were also resistant to ciprofloxacin (MIC 64 µg/mL) and gentamicin (MIC > 64 µg/mL) but remained susceptible to trimethoprim-sulfamethoxazole.

Attempts to transfer resistance to β-lactams by conjugation to *E. coli* J53-2 with the 26 strains tested were unsuccessful. In contrast, transformants were obtained with plasmid DNA of the 19 strains tested by electroporation. The transformants' susceptibility pattern was similar to that of the donor strains, except for ciprofloxacin resistance. Analytical isoelectric focusing showed that all clinical strains and transformants had bands of β-lactamase activity with an alkaline pI of 7.6 and 5.4. Polymerase chain reaction (PCR) amplification of the 26 clinical isolates was positive for bla_CTX-M_ and bla_TEM_ (*7*). The 26 strains of *E. coli* had the same profile by repetitive-element PCR and pulsed-field gel electrophoresis, while unrelated control strains had very different profiles. Sequencing in strains isolated from four of the patients identified a CTX-M-15 β-lactamase and a TEM-1 β-lactamase. The four strains were related to the phylogenetic group B2 and produced the iutA (ferric aerobactin receptor), YuA (*Yersinia* siderophore receptor), and fimH (type I fimbriae) virulence factors (*8*). Incidence of colonization or infection by the culprit strain was 34.3% (12 of 35 patients) within the initial 4-month period and 55.3% (26 of 47 patients) over a 1-year period.

Intensified hygienic procedures implemented in January 2002 contributed to a decrease in the number of cases in February only; since then, a regular increase of new cases extended the outbreak and caused problems with controlling it. All urinary tract infections were successfully treated with a 15-day course of trimethoprim-sulfamethoxazole; however, reinfection occurred in some. Neither incontinence (p = 0.35), dementia (p = 0.22), nor previous antibiotic treatment (amoxicillin, amoxicillin-clavulanic acid, extended-spectrum cephalosporins, and fluoroquinolones [p = 1.00, 0.30, 0.12, 0.52, respectively]) appeared to be risk factors for infection or colonization in our study, but the number of patients is too small to reach a conclusion. However, patients that were infected or colonized had greater functional impairment, especially incontinence and dementia. Nonambulatory status, decubitus ulcers, and feeding tubes were not risk factors for acquiring ESBL-producing *E. coli* in our study.

The outbreak has not been controlled: 13 patients have persistent digestive-tract colonization. Difficulties encountered in controlling such outbreaks may be explained by several factors. Patients cannot be easily isolated in long-term care facilities. Strict isolation and limitation of activity and mobility cannot always be applied because of their impact on social activities.
